# Advanced Methylome Analysis after Bisulfite Deep Sequencing: An Example in *Arabidopsis*

**DOI:** 10.1371/journal.pone.0041528

**Published:** 2012-07-20

**Authors:** Huy Q. Dinh, Manu Dubin, Fritz J. Sedlazeck, Nicole Lettner, Ortrun Mittelsten Scheid, Arndt von Haeseler

**Affiliations:** 1 Max F. Perutz Laboratories, Center for Integrative Bioinformatics Vienna, University of Vienna and Medical University Vienna, Vienna, Austria; 2 Gregor Mendel Institute of Molecular Plant Biology, Austrian Academy of Sciences, Vienna, Austria; Deutsches Krebsforschungszentrum, Germany

## Abstract

Deep sequencing after bisulfite conversion (BS-Seq) is the method of choice to generate whole genome maps of cytosine methylation at single base-pair resolution. Its application to genomic DNA of *Arabidopsis* flower bud tissue resulted in the first complete methylome, determining a methylation rate of 6.7% in this tissue. BS-Seq reads were mapped onto an *in silico* converted reference genome, applying the so-called 3-letter genome method. Here, we present BiSS (Bisufite Sequencing Scorer), a new method applying Smith-Waterman alignment to map bisulfite-converted reads to a reference genome. In addition, we introduce a comprehensive adaptive error estimate that accounts for sequencing errors, erroneous bisulfite conversion and also wrongly mapped reads. The re-analysis of the *Arabidopsis* methylome data with BiSS mapped substantially more reads to the genome. As a result, it determines the methylation status of an extra 10% of cytosines and estimates the methylation rate to be 7.7%. We validated the results by individual traditional bisulfite sequencing for selected genomic regions. In addition to predicting the methylation status of each cytosine, BiSS also provides an estimate of the methylation degree at each genomic site. Thus, BiSS explores BS-Seq data more extensively and provides more information for downstream analysis.

## Introduction

Whole genome sequencing of numerous species and individuals has considerably expanded our understanding of biological diversity and evolution, of normal and abnormal phenotypes. However, it also revealed that regulation of, and differences in, gene expression are not always connected with differences in DNA sequence information. The occurrence of different phenotypes or heritable changes of gene expression, in spite of identical genetic information, has driven the search for additional, epigenetic information transmitted from cell to cell or from parents to progeny. One major component of epigenetic inheritance and regulation is chemical DNA modification by methylation at the 5′ position of cytosine residues (^m^C). This modification occurs in some fungi and insects, in all mammals and higher plants examined to date, and it is sometimes referred to as the fifth base. Research on the role of ^m^C was stimulated by its potential to transmit epigenetic information during DNA replication. Its study was facilitated by the ground-breaking development of bisulfite sequencing, in which non-methylated cytosines get chemically converted into uracil and can be distinguished from methylated residues after PCR amplification and subsequent DNA sequencing [1]. DNA methylation was the first epigenetic mark that could be analysed at high resolution, and its analysis profited substantially from the rapid development of sequencing technologies. It is now accepted as one of the most comprehensive and efficient methods [2]. Bisulfite conversion followed by deep sequencing (BS-Seq) has been successfully applied in many species and cell types to analyze the methylome. However, the “mismatches” after converting unmethylated cytosine residues make the mapping of short reads during BS-Seq more challenging than during genome sequencing. Not for the first time, pioneering epigenetic research was performed in plants, as the first whole methylome was established for *Arabidopsis thaliana* [3], [4]. In addition, some plant genomes have lower levels of total ^m^C compared to that of mammals, therefore more “mismatches” after bisulfite conversion, and more ^m^C in a non-CG context. BS-Seq of genomic DNA isolated from flower buds was fragmented, ligated with methylated adaptors, followed by bisulfite conversion prior to PCR amplification and deep sequencing. The total ^m^C content was calculated as 6.7% of those C positions for which the methylation status could be determined [4]. This is in good agreement with experimentally measured values in the range from 4.6 to 8.6%, obtained by different methods and with different tissue [5]–[7]. However, we noticed a discrepancy between the total ^m^C content calculated after BS-Seq and the frequency estimated from counting cytosines occurring in the raw data from the short-read libraries [4]. Any C in the sequence between the methylated adaptors should directly correspond to methylated cytosines in the genome, complete conversion and low sequence error rates provided. We calculated three Illumina sequencing runs of the data set in [4] to report roughly 10% ^m^C, while the other two suggest 23–26%, probably due to incomplete bisulfite conversion or sequencing errors. Pooling all five runs would correspond to 14.7% methylation. While the discrepancy with previously published values could have been in part due to limited sequencing and unequal coverage, we suspected it to originate mostly from limited mapping of individual short reads to the reference genome since only 78.5% of genomic cytosines were included in at least 2 mapped reads [4]. This could have been due to the mapping procedure: the so-called three-letter genome method, in which all genomic cytosines are converted *in silico* to thymine, before the reads are mapped using ELAND –software from the Illumina company, interpreting C-T mismatches as indicative for methylated cytosines during the downstream analysis [4]. We refer to it as A3M (*Arabidopsis* 3-letter Methylome) method in the following.

Aiming to improve mapping efficiency and accuracy for analysis of plant material, we have developed BiSS (**Bi**sulfite **S**equence **S**corer), based on an efficient Smith-Waterman (SW) local alignment implementation for BS-Seq mapping with a customized alignment scoring function. SW has the potential to produce superior alignments due to the base-by-base resolution in sequence comparison. Previous [8] and our own recent work (Sedlazeck et al, 2012, in revision) suggests that SW local alignment is in fact the most sensitive method for next-generation sequencing mapping available to date. High specificity and confidence are obtained with this method, admittedly at the cost of increased computing time. SW local alignment was implemented in the MAQ program recently [9], [10] but not yet evaluated in comparison with other methods. Therefore, we applied this SW approach using a special asymmetric score for BS-Seq data to re-analyze the *Arabidopsis* methylome data set with BiSS.

For the data analysis downstream of mapping the reads, we introduced a comprehensive adaptive error estimate that accounts for sequencing errors, erroneous bisulfite conversion and also wrongly mapped reads. With BiSS, we were able to map many more short reads unambiguously to the reference genome than other methods. The increased coverage gives increased power to call an individual cytosine methylated or un-methylated, thus allowing the determination of methylation status at significantly more sites. The re-analysis of the *Arabidopsis* methylome dataset using BiSS and the adaptive error estimate could identify the methylation status of an extra 10% of genomic cytosines and resulted in estimation for the global methylation to be 7.7% of all cytosines. We validated these results by traditional individual traditional bisulfite sequencing (ITBS) at several genomic regions with discrepancy. In all but one locus these results confirmed the prediction from the BiSS analysis. Moreover, these data show that the BiSS method provides an accurate estimation of the degree of methylation at individual partially methylated genomic sites.

## Results

### BiSS can map more reads unambiguously to the reference genome

BiSS calls a read uniquely mapped if it can identify only one SW-alignment with the highest score. To avoid mapping artefacts we excluded reads with an alignment identity (not considering bisulfite mismatches) below 85% within the aligned region. More than half (53.2%) of the raw reads were above this threshold and were used for the downstream analysis. In total, we were able to map approximately 77 million unique reads, 1.96×times more than the A3M approach used in the original data analysis. There are also several other published methods to analyze BS-seq data, such as BSMAP [11], RMAPBS [12], BRAT [13], BS-Seeker [9], PASH 3.0 [14], BisMark [15], and MethylCoder [16]. We compared BiSS to a selection of these, including the most recently published aligners BisMark [15] and BSMAP [11], the most sensitive mapping according to previous comparative studies [2], [17]. With the parameters recommended by the authors [11], BSMAP mapped 1.60 times less reads than BiSS (Table 1). RMAP mapped 1.61 times less reads, and all other methods performed in a comparable range or less (Table 1). To compare the BiSS-generated *Arabidopsis* methylome with the previous interpretation of the same data, we chose A3M for a more detailed comparison, since this was applied in the pioneering approach to generate a single-bp methylation profile after mapping. Details on the comparison and mapping statistics can be found in Tables S1, s2, S3, S4, S5. In summary, BiSS almost doubles the number of mapped reads that can be used for ^m^C analysis. This translated to over 10% more cytosines in the genome that are covered by at least 2 mapped reads, the minimum required for methylation calling in the A3M approach.

**Table 1:** Comparison of mapping results for BiSS and selected other aligning programs^1^.

	Number of mapped reads	Number of analyzed reads (Uniquely mapping, except BiSS)
**A3M** 1	55,805,931 (38.6%)	39,113,599 (27.1%)
**BSMAP** 2	73,215,737 (50.7%)	47,922,346 (33.2%)
**RMAP** 3	64,061,732 (44.4%)	47,859,115 (33.1%)
**BS-Seeker**	51,657,927 (35.8%)	37,939,172 (26.3%)
**BisMark**	50,324,319 (34.8%)	37,706,400 (26.1%)
**BiSS**	103,073,409 (71.4%) (Uniquely highest scored)	76,841,502 (53.2%) (> = 85% identity)

Default parameters unless otherwise specified.

1 A3M – results reported by [4].

2 BSMAPv1 parameters: -p 8 -s 12 -r 2 -w 100 -n 1 -v 5 -g 5, recommended by the authors, maximal 5 mismatches.

3 RMAP parameters: -m 5 –v, default parameters.

### BiSS extends the methylome of Arabidopsis thaliana

Since the number of Cs for which the methylation status could be assigned differs between the two methods, we investigated the degree of overlap between them (Table 2). There was good agreement (76.5%) between the two methods when classifying methylated (M/M) and unmethylated (U/U) cytosines. For 9.9% of cytosines neither A3M nor BiSS could make a call (X/X). However, BiSS was able to determine the methylation status of 89.5% of the genomic Cs, in contrast to 79% for A3M. In total, BiSS called 6.9% of all Cs methylated (Table 2), 30% more than A3M, which scored 5.3% methylated. The additional Cs called methylated by BiSS were mainly from the fraction where A3M was unable to make a call (X in Table 2). However, some Cs (0.9%) called unmethylated by A3M were assigned to the methylated category by BiSS (M/U). A substantial fraction (9.4%) of Cs, for which A3M could not call the methylation state, was assigned to the unmethylated category by BiSS (U/X). Small shifts also occur in the opposite directions: 0.9% A3M-called methylated Cs are considered unmethylated by BiSS (U/M), and only 0.6% of Cs not determined by BiSS were assigned by A3M (X/M and X/U, Table 2). Thus, the more efficient mapping procedure employed in BiSS was able to considerably reduce the uncharted portion of the methylome. In summary, the analysis of the data set by BiSS largely corroborates the previously published analysis on the amount and distribution of genomic ^m^Cs but was able to determine the methylation status at 10.6% more sites in the reference genome. This reanalysis indicates higher levels of ^m^C (7.7%) in flower tissue than previously reported (6.7%).

**Table 2:** Congruency between methylation calling by A3M and BiSS.

		A3M		
		M (5.3%)	U (73.6%)	X (21.1%)
**BiSS**	**M (6.9%)**	1,839,780 (4.3%) **M/M**	371,418 (0.9%) **M/U**	749,156 (1.7%) **M/X**
	**U (82.6%)**	397,308 (0.9%) **U/M**	30,970,572 (72.2%) **U/U**	4,028,624 (9.4%) **U/X**
	**X (10.5%)**	30,359 (0.07%) **X/M**	230,988 (0.54%) **X/U**	4,257,806 (9.9%) **X/X**

M: methylated, U: unmethylated, X: not determined due to lack of sufficient sequencing coverage. Percentages refer to the total number of genomic cytosines.

**Figure 1:**
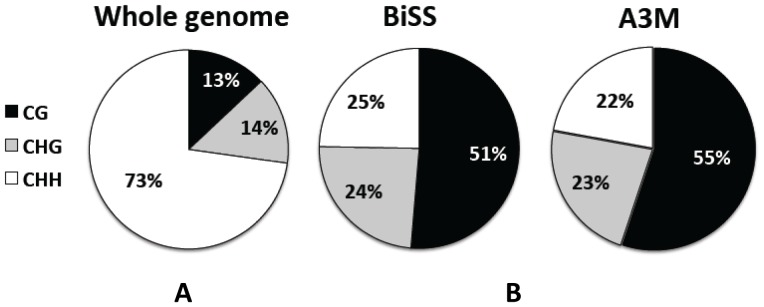
Distribution of cytosine sequence context. (A) Frequency of sequence context in the reference genome. (B) Frequency of sequence context of methylated C according to BiSS and A3M.

To gain a deeper insight into the different performance of both methylation assignment approaches, we computed the differences corresponding to the sequence context of the cytosines (CG, CHG, CHH; with H = A, C, or T). The results are illustrated in Figure 1. In the reference genome, CHH is naturally most frequent (73%), followed by CHG and CG, the latter occurring at almost equal frequency (Figure 1A). The frequency distribution of ^m^C with respect to the sequence context shows a strong preference for ^m^CG as expected, and is nearly identical for A3M and BiSS (Figure 1B). Thus, although BiSS assigns a methylation status to more genomic Cs, it does it without a bias for any sequence context.

**Figure 2:**
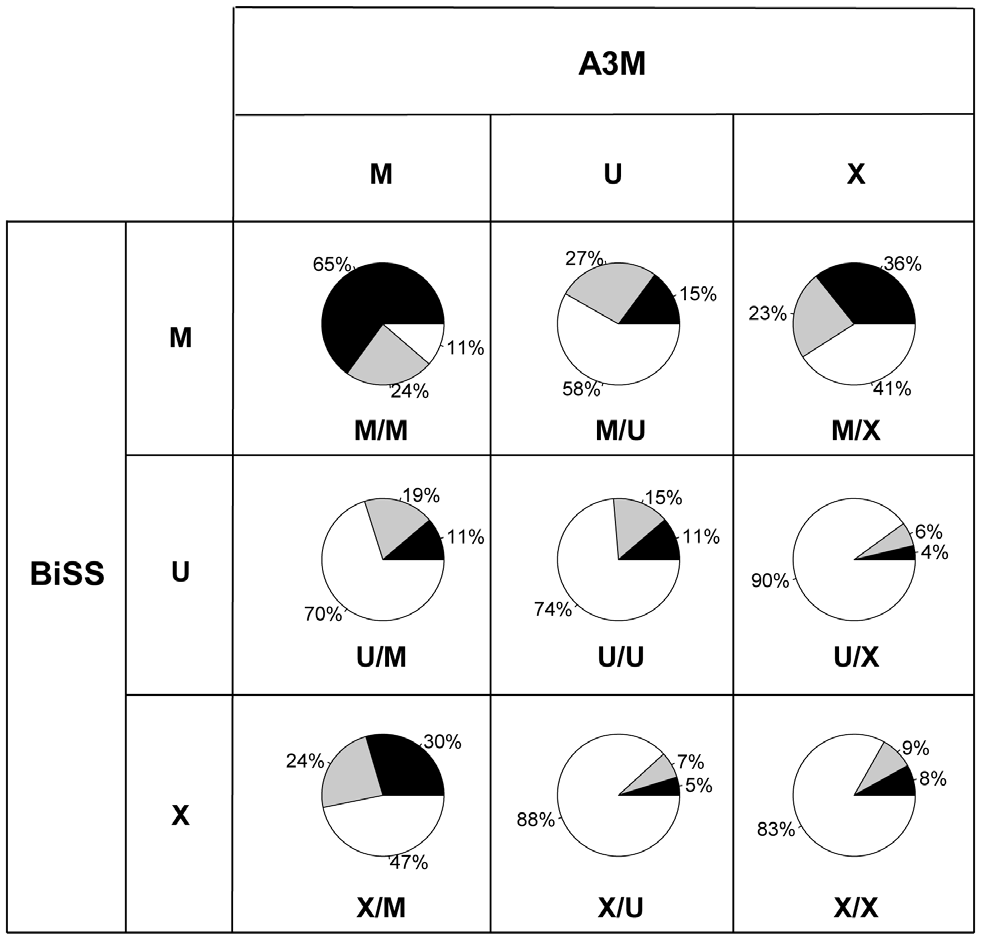
Methylation status according to BiSS and A3M split into distribution of cytosine sequence context. M, methylated; U, unmethylated; X, not determined. Percentages refer to the total numbers in Table 2.

We have further split the congruency assignment (Table 2) into the C-sequence context (Figure 2). Sixty five per cent of methylated Cs identified by both methods (M/M) occur in a CG dinucleotide context. The frequencies of C-contexts for unmethylated Cs (U/U) are almost identical to their genomic frequencies. The methylated Cs only called by BiSS (M/X) were in all sequence contexts, while unmethylated Cs only called by BiSS (U/X) occurred largely in the CHH context (Figure 2). The few Cs only called by A3M have a similar distribution. Taken together with the absolute numbers in Table 2, it can be concluded that the SW scoring method used by BiSS is able to assign a methylation status to a significant number of CHH sites that could not be called by A3M. This suggests that the CHH context is more challenging to map, as seen from the large fraction of CHH sites where the two methods either disagree or both fail to make a call.

### Evaluating the methylation level

The decision to call a genomic C as methylated is based on a statistical test that considers the number of reads mapped to a genomic C, the C/T counts at the site and the estimated adaptive error (see Material and Methods). Thus, a genomic C can be called methylated with confidence even if not every mapped read contains a C at that site. The coexistence of Cs and Ts at individual positions reflects the biologically well-known heterogeneity of methylation between alleles in the same genome or in different cell types, tissues or individuals.

**Figure 3:**
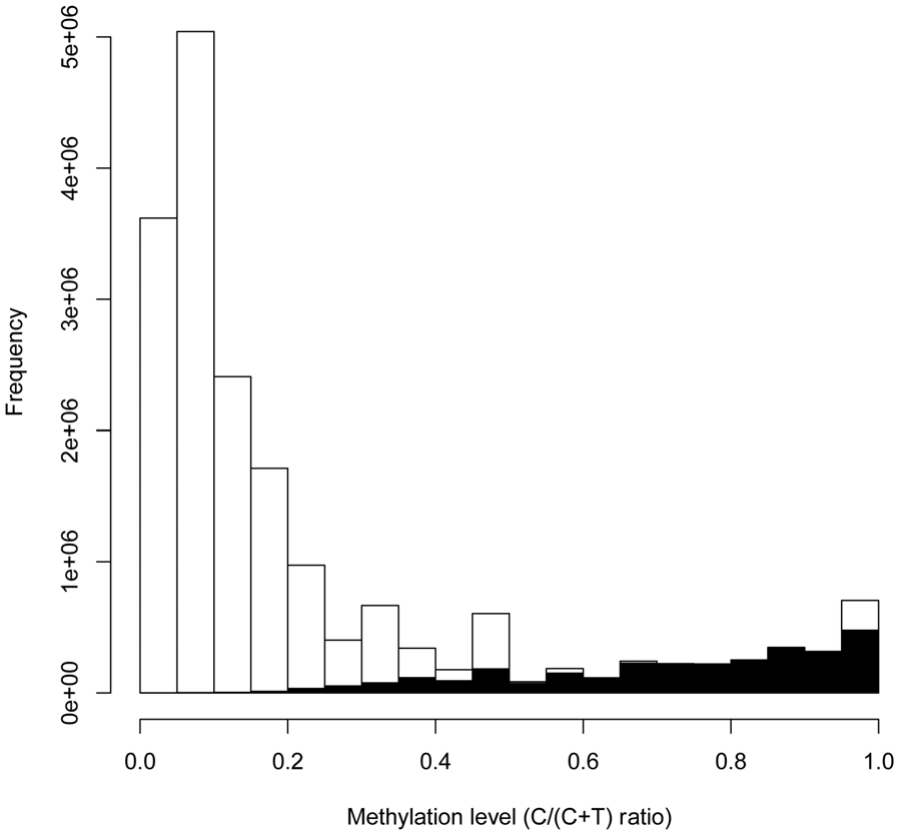
Global methylation level. Ratio of the number of mapped Cs divided by the number of mapped C plus T for all classified Cs. Black: Cs that are called methylated, White: C that are not called methylated.

A plot of the degree of ^m^C, calculated as the C/(C+T) ratio of mapped reads for each C in the reference genome shows that this ratio varies across the entire range from 0–1 (Figure 3). For the majority of the Cs that BiSS calls methylated, the ratio is typically above or equal to 0.5. However, 1.1% of the ^m^Cs in the genome display a C/(C+T) less than 0.5. This suggests that genomic Cs with a C/(C+T) ratio larger than 0.4, are probably methylated but were not called as such due to the very conservative nature of the test. Thus our estimate of 7.7% methylated Cs is likely to be an underestimate, and the true methylation level may be higher (for example, if all Cs with a C/(C+T) above 0.4 are called methylated we get an estimate of 9.1%.

### BiSS methylation calling is validated by independent bisulfite sequencing

**Figure 4:**
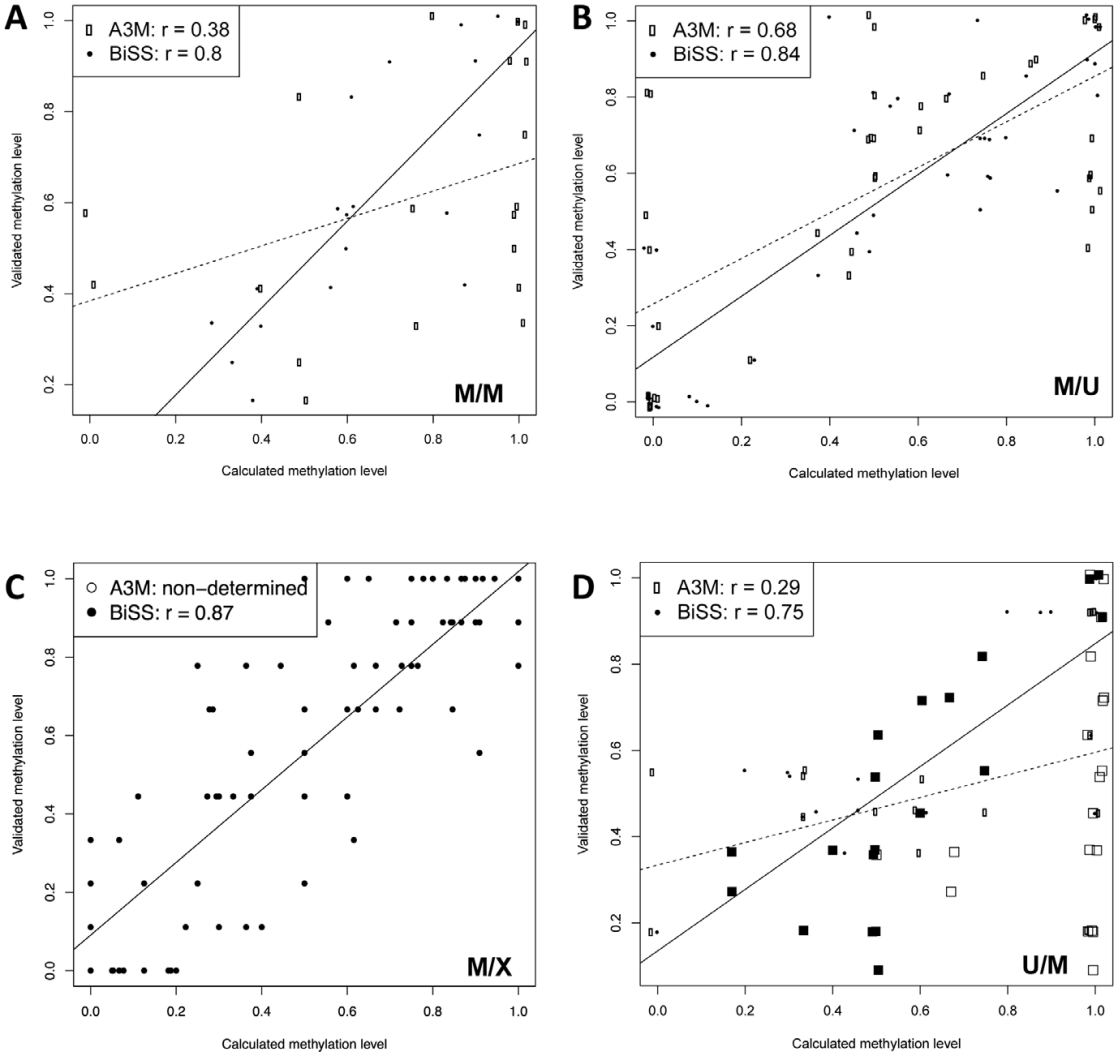
Examples for validation by individual bisulfite sequencing. The plots show the correlation between calculated and validated methylation levels (C/(C+T)) from regions selected for congruency (A) or disagreement (B–D) between BiSS and A3M. Each point represents one cytosine position. The x-axis corresponds to the methylation levels calculated from either BiSS (filled circles and black regression lines) or A3M (open circles and dotted regression lines); the y-axis shows the result of individual bisulfite sequencing. The legends show the Pearson correlation coefficients. (A) Methylated region according to both methods (M/M). (B) A region called methylated by BiSS but not by A3M (M/U); the rectangles indicate experimentally validated Cs congruent to BiSS (filled) and discrepant to A3M (open). (C) A region called methylated by BiSS but not by A3M due to insufficient sequencing coverage (M/X). (D) A region called methylated by A3M but not by BiSS (U/M), the rectangle symbols are the same as in (B).

To confirm the improved accuracy of the BiSS method compared to A3M, methylation levels at selected regions of the genome were independently determined by individual traditional bisulfite sequencing (ITBS) and compared to the results from BiSS and A3M. We selected 2 regions where both methods reported to have high methylation (M/M) and two regions were both reported no methylation (U/U). We further identified 4 regions where the two methods disagreed in methylation calling (2×M/U and 2×U/M), and 6 regions that were mapped by BiSS but lacked sufficient sequencing coverage in A3M results (M/X and U/X). The regions represent genic, intergenic and repetitive sequences (Table S22). For each region, we compute the Pearson correlation coefficient between calculated methylation levels from A3M/BiSS and ITBS. Representative correlations for each comparison category are shown in Figure 4.

The BiSS/A3M methylation levels were confirmed for the U/U and M/M regions, Figure 4A, (Figure S1A, S3 and Tables S6, S7, S8, S9). However the BiSS prediction had a higher correlation with the ITBS data, even if both methods call high methylation. For one of the two regions of the M/U category (Figure 4B, Table S10), BiSS, but not A3M, results were in good agreement with the ITBS data. However, at the second M/U region the BiSS results did not agree with the ITBS data (Figure S1B, Table S11). This appears to be because there were only 1–2 sequencing runs on which BiSS based the methylation call. The low coverage in this region is likely due to having only a few cytosines, exclusively in CHH context, which are more difficult to map. However, even at this locus the correlation is not much different for both methods (r = 0.37 for A3M and r = 0.36 for BiSS). Apart from this exception, there was good agreement between BiSS results and Sanger data for regions where Cs could not be classified by A3M. One example is shown in Figure 4C, five others in the Figure S2 and Tables S12, S13, S14, S15, S16, S17. The U/M regions classified by A3M as highly methylated but categorized by BiSS as unmethylated were indeed unmethylated according to ITBS. The reason for better performance of BiSS appears to be due to either run-specific information (Figure 4D, Table S18) or simply by gaining higher coverage (Figure S1C, Table S19). Thus, the BiSS predictions had a higher correlation with Sanger data compared to A3M not only for the methylation calling but also for predicting the level of methylation.

An interesting case was a region called methylated by A3M (with fully methylated for many individual sites), which was in strong disagreement with the ITBS data, with a negative correlation coefficient. The BiSS results had a high correlation with the ITBS data but did not call any methylation there (Figure S4 and Table S20). A closer look revealed that the A3M-determined methylation level was based on a read coverage of only 2. Notably, this indicates that we still underestimate the methylation rate due to the very conservative test mentioned above (Figure 3). Thus, BiSS could obtain a higher coverage and indicated rather low methylation here. This supports the notion that BiSS can provide a more realistic interpretation of the BS-Seq data, especially in regions where A3M suffers from low coverage, and at many CHH sites.

In summary, BiSS can help to improve mapping of BS-Seq reads to the reference genome, to provide higher coverage, and to provide a refined and more accurate methylome map.

## Discussion

BiSS, a scoring method for whole genome bisulfite deep sequencing data, takes advantages of SW alignment to evaluate the bisulfite conversion as an add-on for the general SW-based mapping package (NextGenMap, Sedlazeck et al, 2012, in revision). In addition, BiSS incorporates an adaptive error into the binomial test to correct for the mismatch ratio including sequencing or mapping errors in the downstream analysis. Moreover, BiSS also exploits the potential of considering run-specific information, which can reduce the effect of errors introduced by sequencing bias. It also allows the separate analysis of individual sequencing runs representing biological replicates.

The re-analysis of previously published BS-Seq data from *Arabidopsis* by BiSS increased the number of cytosines for which the methylation status could be reliably determined by 10%, largely due to higher mapping efficiency. In particular, BiSS successfully identified the methylation status at a significant number of CHH-context cytosines, where the A3M method performs poorly. Independent bisulfite sequencing confirmed the BiSS predictions at regions where it disagreed with the A3M method. It also confirmed that BiSS more accurately predicted the level of methylation at partially methylated cytosines. Thus, BiSS provides a new and more accurate reference for the floral *Arabidopsis* methylome.

We note that some researchers prefer to trim or filter reads prior to the alignment step to remove bases with low quality scores. To test if filtering prior to alignment affected the performance of BiSS we repeated the alignment after filtering the raw reads using the FASTX toolkit [http://hannonlab.cshl.edu/fastx_toolkit/index.html] When mapping this filtered data set of 107 Mio (71%) reads, BiSS was still able to map 10% more reads than BSMAP, the best performing of the published aligners. BiSS could also be applied to other *Arabidopsis* methylome datasets obtained from different material [3], [18], and to data from human DNA in the same order of magnitude in term of running times compared to existing methods (Table S21).

The improved performance of BiSS with respect to the number of mapped reads for methylation analysis is mainly due to the SW-based mapping method, applied here to bisulfite deep sequencing data in open-source software. The algorithm compares subsequence of different lengths and thereby optimizes the similarity detection, compared to other BS-Seq mapping methods, which either encode the reference genome in a three letters alphabet or use special bisulfite conversion masks for mapping the reads with general-purpose software. SW alignment has also the ability to stop aligning, if the reads get too different, due to increasing sequencing errors towards the end of longer NGS reads.

In attempting to maximize the number of aligned reads, one runs the risk of generating a data set containing a significant portion of incorrect alignments. Therefore, we independently validated the ^m^C frequency at selected genomic regions (Figure 4), with results that excluded this possibility (Table S21). Naturally, the SW-based method requires extra computing time (Table S21) compared to other methods. However, in many cases the gain of extra mapping information will outweigh this disadvantage. As long as the costs of next generation sequencing remain an issue, at least for researchers outside of large genome centres, it is reasonable to apply optimized evaluation methods. BiSS can also align both single & paired-end as is described in the manual. Our results suggest that the improved performance of BiSS compared to competing methods is in large part due to its superior ability to assign the methylation status to cytosines outside of CG context. This is sure to be appreciated by some researchers, given the growing evidence for ^m^CHG and ^m^CHH in specialized mammalian cells [19].

## Methods

### (Experimental and computational procedures)

#### Mapping deep sequencing reads after bisulfite conversion

BiSS uses the SW local alignment to map the sequencing reads after bisulfite conversion (BS-reads) to the reference genome with a special scoring function. To speed up computation, a hash-table stores the positions of all k letter words (k-mers) in the genome. The k-mers are encoded as numbers (keys) as follows: Nucleotide A is converted into 00, C to 01, G to 10, and T to 11. Thus an 8-mer results in a string of 16 zeros or ones; this string can be converted into an integer number that serves as key to point to the genomic positions. Keys were also computed for all k-mers in a BS-read. Together with the hash-table, the BS-read keys allow a quick retrieval of the genomic positions in the reference genome, where read and genome share the same k-mers.

**Figure 5:**
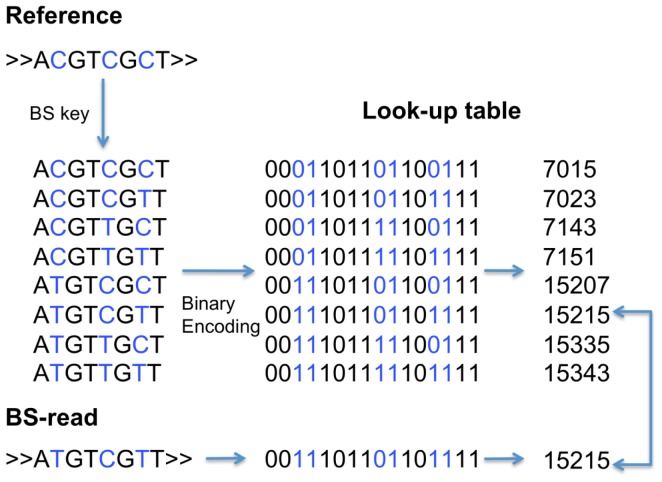
Example for an asymmetric look-up table for 8 k-mers. The 8-mer ACGTCGCT (corresponds to key 7015) generates 7 other keys. The 8-mer ATGTCGTT (key 15215) from the BS-read can be looked up to find its referenced key as ACGTCGCT (key 7015), ACGTCGTT (key 7023), ATGTCGCT (key 15207) and ATGTCGTT (key 15215) but no others.

Because bisulfite conversion turns unmethylated Cs into Ts, the 1-to-1 correspondence between k-mer in a read and in the reference genome is lost. To account for this, a pre-computed look-up table was generated that for each k-mer stores the alternative keys that can be computed by switching a C into a T. Figure 5 exemplifies this for the 8-mer A_1_C_2_G_3_T_4_C_5_G_6_C_7_T_8_ (key 7015), switching C_7_ into a T provides key 7023 and so on. Thus, the k-mer A_1_T_2_G_3_T_4_C_5_G_6_T_7_T_8_ with key 15215 from a BS-read will automatically be associated with the potential genomic regions from the additional keys in the look-up table. The look-up table needs to be computed only once, thus saving computing time. In this study k was set to 12.

The hashing table is searched for k-mer co-occurring in a BS-read and the reference genome to determine the potential locations of SW alignment. To reduce the number of potentially matching genomic regions that need to be scored, at least two k-mers in the BS-read must occur in close proximity in the reference genome. Moreover, we allow that the distance between two neighbouring k-mers in the read and their distance in the corresponding genomic sequence can differ by 3 nucleotides. Finally, to reduce the number of unspecific matches, we excluded all 12-mers from hashing that contain 8 or more Ts. The parameter 8 is the default parameter that can be specified by the user. For these reads, the SW algorithm is applied with a special scoring function, specifically a score 4 for match (including C-T mismatch, where C occurs in the reference genome and G-A in other strand), −2 for mismatch, −10 as gap penalty. The genomic region providing the highest alignment score is considered as the genomic origin of the read. To cope with the huge number of SW alignment computation the package NextGenMap (Sedlazeck et al, 2012, in revision) which implements a banded SW algorithm speeded up by Graphical Processing Unit (GPU) computing was used.

### Re-analyzing the *Arabidopsis thaliana* methylome

BiSS was used to re-analyze the BS-read data of *Arabidopsis thaliana Col-0* wild type generated by [4]. This read library consists of 5 Illumina runs with approximately 150 million 56-bp BS-reads, thus providing a theoretical coverage of roughly 56. After mapping the reads, only the uniquely mapped reads with at least 85% similarity (calculated from local read-reference alignment after excluding C-T mismatches on the Watson strand and G-A mismatches on the Crick strand) were further analyzed. Following [4], only genomic cytosines with at least 2 mapped reads are further used for statistical calling of the methylation status (see below).

To compare BiSS results with those of A3M, the same assembly version (TAIR7 *Arabidopsis thaliana*) was used as a reference. The alignment profile of the A3M method was downloaded from NCBI Gene Expression Omnibus (accession number GSE10877), the list of methylcytosines was provided by the authors of [4].

### Methylcytosine calling

To determine if a specific cytosine was methylated a binomial test was performed. The parameters of the binomial distribution are *n* as the coverage at a genomic cytosine position, *p* = 0.04 as the assumed sequencing or conversion error, and *m* as the number of BS-reads that carry a C at that position, indicating methylation. In addition, a so-called adaptive error was introduced. Whenever the frequency of non C-T mismatches at a given genomic cytosine position is bigger than the assumed sequencing/conversion error, we used the local mismatch frequency of read alignment as site specific error rate for the binomial distribution. Thus, the adaptive error will reduce a potential bias due to reads that are aligned to the wrong genomic region. To account for multiple testing, the False Discovery Rate p-value adjustment based on Benjamini and Hochberg [20] from the R statistical computing package (www.R-project.org) was used.

The actual methylcytosine calling was done in several steps:

First the pool of all mapped reads from the 5 sequencing runs was considered to identify genomic Cs with a read coverage of at least 2. Then a binomial test was applied as described above, together with the FDR correction, to test the methylated cytosines with significance cut-off of 5%. The resulting list of methylated cytosines was then analyzed to account for differences between sequencing runs. The binomial test was then applied for all mapped reads from individual runs, again requiring coverage of at least two. If the majority of runs where the test could be performed suggested methylcytosine, the genomic C was called as methylated, otherwise not. This approach considers the experiments with varying bisulfite conversion rates in different runs. In case of no sufficient coverage in any individual run, the methylation decision was based on the global test in the pooled set.

### Experimental validation

A window-scanning strategy was used to identify genomic regions of 250–500 bp (at least 30% C-content) for which the BiSS and A3M methods were in (dis-)agreement as to the extent of calculated methylation. These sequences were analysed for their methylation level by conventional bisulfite sequencing of individual regions. For this, plants of the *Arabidopsis thaliana* accession Col-0 (Columbia) were grown under long day (16 h/d) light condition at 21°C and DNA was extracted from 100 mg of unopened flower buds using the Phytopure DNA extraction kit (GE Healthcare; Little Chalfont, UK). After an additional RNase A treatment, 1 µg of DNA was digested with either *Eco*RI or *Kpn*I (excluding a restriction recognition site between the primers for the region) and purified with a PCR purification kit (Qiagen; Hilden, Germany) according to the manufacturers' protocol. Five hundred ng of DNA were bisulfite-converted using the EpiTect Bisulfite Kit (Qiagen; Hilden, Germany) according to the manufacturers' alternative protocol for dilute solutions. The sequences of interest were amplified using the polymerase PfuTurbo Cx (Agilent Technologies; Santa Clara, CA) and methylation-neutral primers. Amplicons were cloned into the pJET1.2/blunt vector (Fermentas; Vilnius, Lithuania) and transformed into E. coli. For each amplicon at least 8 independent clones were sequenced, aligned and analysed as described in [21].

### Data access

The BiSS analysis pipeline is based on Graphic Processing Unit computation on CUDA (Computer Unified Device Architecture) framework, and details of the *Arabidopsis* methylome generated by BiSS are available at http://www.cibiv.at/software/ngm/BiSS.

System Requirements: CPU: SSE enabled dual-core (quad-core recommended), RAM: 4 GB (16 GB recommended), GPU (optional): CUDA (Nvidia) or ATI Stream Technology (ATI) enabled, OS: Linux (OpenSUSE with gcc 4.3.4 recommended), Software: CUDA 3.2 (or higher), AMD Accelerated Parallel Processing SDK 2.5.

## Supporting Information

Figure S1 **Validation by individual bisulfite sequencing.** The plots show the correlation between calculated and validated methylation levels (C/(C+T)) from regions selected for congruency (A) or disagreement (B–C) between BiSS and A3M. Each point represents one cytosine position. The x-axis corresponds to the methylation levels calculated from either BiSS (filled circles and black regression lines) or A3M (open circles and dotted regression lines); the y-axis shows the result of individual bisulfite sequencing. The legends show the Pearson correlation coefficients. (A) Methylated region according to both methods (M/M). (B) A region called methylated by BiSS but not by A3M (M/U); the rectangles indicate experimentally validated Cs congruent to BiSS (filled) and discrepant to A3M (open). (C) A region called unmethylated by BiSS but methylated by A3M (U/M); the rectangle symbols are the same as in (B).
(PDF)

Figure S2 **Validation by individual bisulfite sequencing.** The plots show the correlation between calculated and validated methylation levels (C/(C+T)) from 5 different regions (A–E) selected for methylation calling by BiSS versus undetermined state by A3M. Each point represents one cytosine position. The x-axis corresponds to the methylation levels calculated from BiSS; the y-axis shows the result of individual bisulfite sequencing. The legends show the Pearson correlation coefficients.
(PDF)

Figure S3 **Validation by individual bisulfite sequencing.** The plot shows the correlation between calculated and validated methylation levels (C/(C+T)) from a region selected for disagreement between BiSS (calling it unmethylated) and A3M (calling it methylated). Each point represents one cytosine position. The x-axis corresponds to the methylation levels calculated from either BiSS (filled circles and black regression lines) or A3M (open circles and dotted regression lines); the y-axis shows the result of individual bisulfite sequencing. The legends show the Pearson correlation coefficients.
(PDF)

Figure S4 **Validation by individual bisulfite sequencing.** The plot shows the correlation between calculated and validated methylation levels (C/(C+T)) from a region selected for disagreement between BiSS (calling it unmethylated) and A3M (calling it methylated). Each point represents one cytosine position. The x-axis corresponds to the methylation levels calculated from either BiSS (filled circles and black regression lines) or A3M (open circles and dotted regression lines); the y-axis shows the result of individual bisulfite sequencing. The legends show the Pearson correlation coefficients.
(PDF)

Table S1 **Number of reads with given mismatches.**
(XLS)

Table S2 **Number of reads with given alignment length and identity.**
(XLS)

Table S3 **Mapping statistics.**
(XLS)

Table S4 **Methylcytosine calling statistics.**
(XLS)

Table S5 **C-context methylation statistics.**
(XLS)

Table S6 **Characteristics of validated regions (M/M category).**
(XLS)

Table S7 **Characteristics of validated regions (M/M category).**
(XLS)

Table S8 **Characteristics of validated regions (M/U category).**
(XLS)

Table S9 **Characteristics of validated regions (M/U category).**
(XLS)

Table S10 **Characteristics of validated regions (U/M category).**
(XLS)

Table S11 **Characteristics of validated regions (U/M category).**
(XLS)

Table S12 **Characteristics of validated regions (U/U category).**
(XLS)

Table S13 **Characteristics of validated regions (U/U category).**
(XLS)

Table S14 **Characteristics of validated regions (M/X category).**
(XLS)

Table S15 **Characteristics of validated regions (M/X category).**
(XLS)

Table S16 **Characteristics of validated regions (M/X category).**
(XLS)

Table S17 **Characteristics of validated regions (M/X category).**
(XLS)

Table S18 **Characteristics of validated regions (M/X category).**
(XLS)

Table S19 **Characteristics of validated regions (M/X category).**
(XLS)

Table S20 **Characteristics of validated region with underestimated methylation.**
(XLS)

Table S21 **Comparison with simulated data (Arabidopsis, Human).**
(XLS)

Table S22 **Annotation of validated regions.**
(XLS)
