# Supplementary material for: Advanced Methylome Analysis after Bisulfite Deep Sequencing: An Example in Arabidopsis
Source: PLoS One. 2012 Jul 20;7(7):e41528. doi: 10.1371/journal.pone.0041528 (PMC3401099; doi:10.1371/journal.pone.0041528)
Supplement: Figure S1 — Validation by individual bisulfite sequencing. The plots show the correlation between calculated and validated methylation levels (C/(C+T)) from regions selected for congruency (A) or disagreement (B–C) between BiSS and A3M. Each point represents one cytosine position. The x-axis corresponds to the methylation levels calculated from either BiSS (filled circles and black regression lines) or A3M (open circles and dotted regression lines); the y-axis shows the result of individual bisulfite sequencing. The legends show the Pearson correlation coefficients. (A) Methylated region according to both methods (M/M). (B) A region called methylated by BiSS but not by A3M (M/U); the rectangles indicate experimentally validated Cs congruent to BiSS (filled) and discrepant to A3M (open). (C) A region called unmethylated by BiSS but methylated by A3M (U/M); the rectangle symbols are the same as in (B). (PDF) [file pone.0041528.s001.pdf]

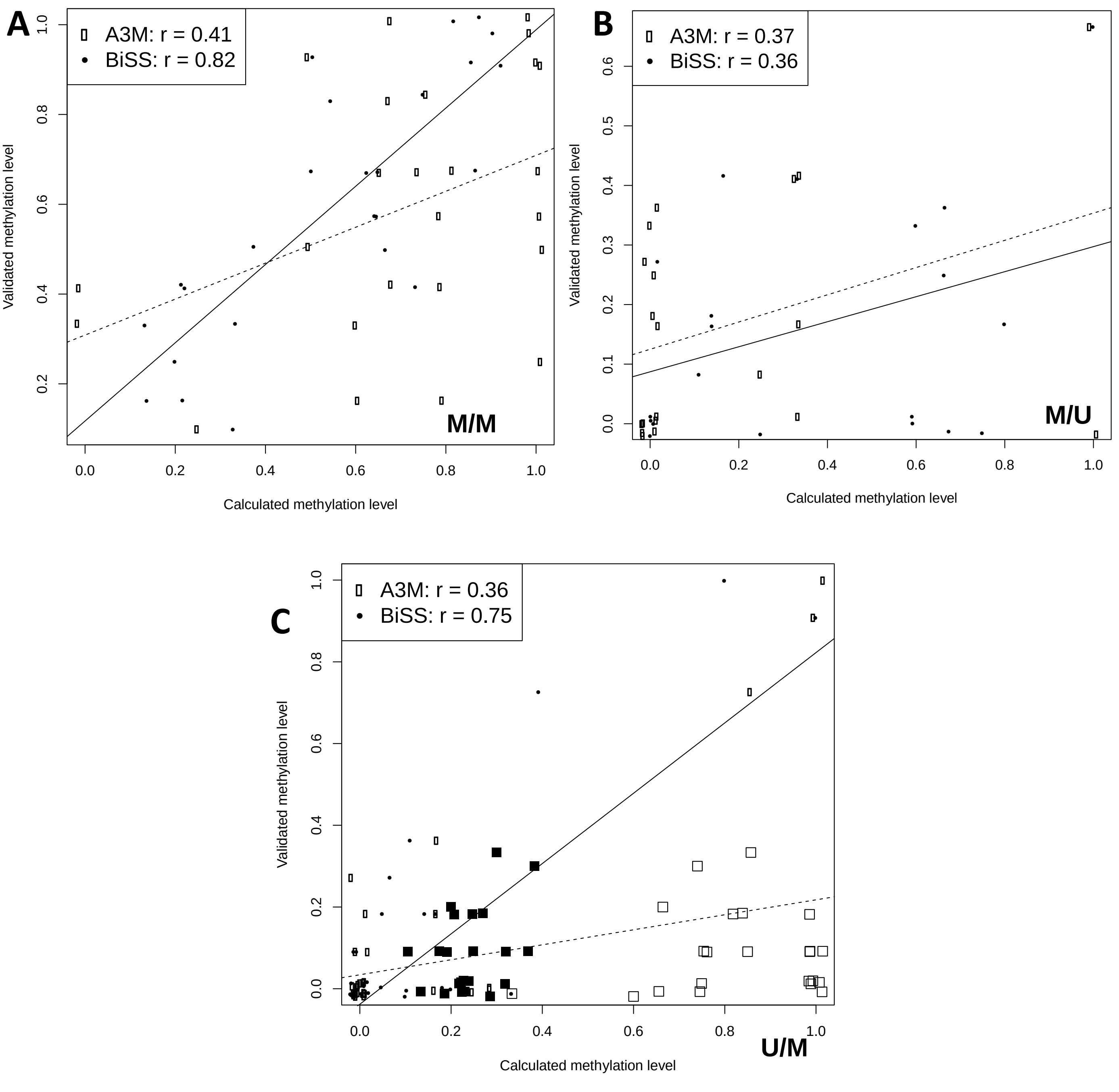

Dinh et al. Supplementary Figure 1

Validation by individual bisulfite sequencing. The plots show the correlation between calculated and validated methylation levels (C/(C+T)) from regions selected for congruency (A) or disagreement (B-C) between BiSS and A3M. Each point represents one cytosine position. The x-axis corresponds to the methylation levels calculated from either BiSS (filled circles and black regression lines) or A3M (open circles and dotted regression lines); the y-axis shows the result of individual bisulfite sequencing. The legends show the Pearson correlation coefficients. (A) Methylated region according to both methods (M/M). (B) A region called methylated by BiSS but not by A3M (M/U); the rectangles indicate experimentally validated Cs congruent to BiSS (filled) and discrepant to A3M (open). (C) A region called unmethylated by BiSS but methylated by A3M (U/M); the rectangle symbols are the same as in (B).
