# Supplementary material for: Advanced Methylome Analysis after Bisulfite Deep Sequencing: An Example in Arabidopsis
Source: PLoS One. 2012 Jul 20;7(7):e41528. doi: 10.1371/journal.pone.0041528 (PMC3401099; doi:10.1371/journal.pone.0041528)
Supplement: Figure S2 — Validation by individual bisulfite sequencing. The plots show the correlation between calculated and validated methylation levels (C/(C+T)) from 5 different regions (A–E) selected for methylation calling by BiSS versus undetermined state by A3M. Each point represents one cytosine position. The x-axis corresponds to the methylation levels calculated from BiSS; the y-axis shows the result of individual bisulfite sequencing. The legends show the Pearson correlation coefficients. (PDF) [file pone.0041528.s002.pdf]

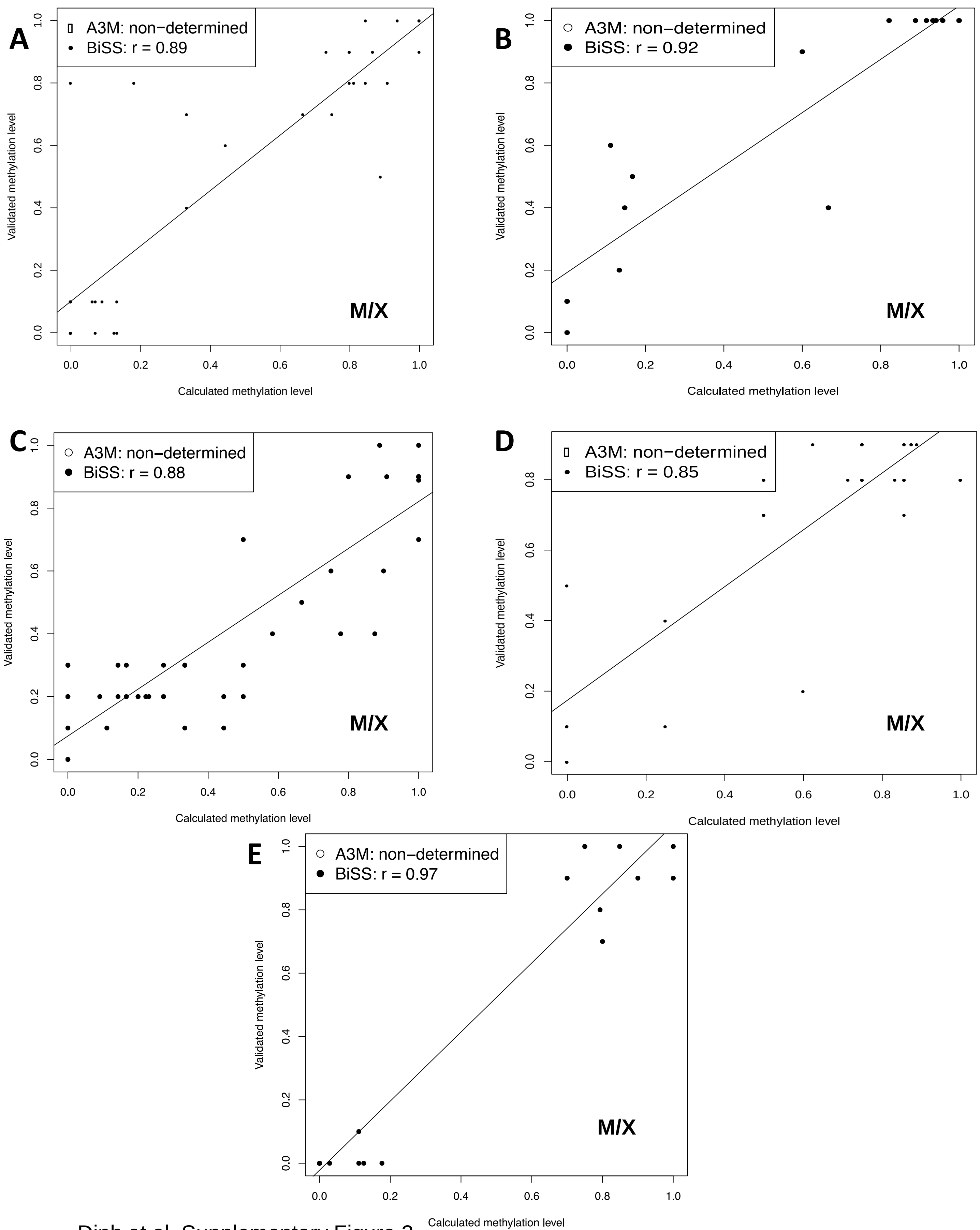

Dinh et al. Supplementary Figure 2

Validation by individual bisulfite sequencing. The plots show the correlation between calculated and validated methylation levels (C/(C+T)) from 5 different regions (A-E) selected for methylation calling by BiSS versus undetermined state by A3M. Each point represents one cytosine position. The x-axis corresponds to the methylation levels calculated from BiSS; the y-axis shows the result of individual bisulfite sequencing. The legends show the Pearson correlation coefficients.
