# Supplementary material for: Advanced Methylome Analysis after Bisulfite Deep Sequencing: An Example in Arabidopsis
Source: PLoS One. 2012 Jul 20;7(7):e41528. doi: 10.1371/journal.pone.0041528 (PMC3401099; doi:10.1371/journal.pone.0041528)
Supplement: Figure S3 — Validation by individual bisulfite sequencing. The plot shows the correlation between calculated and validated methylation levels (C/(C+T)) from a region selected for disagreement between BiSS (calling it unmethylated) and A3M (calling it methylated). Each point represents one cytosine position. The x-axis corresponds to the methylation levels calculated from either BiSS (filled circles and black regression lines) or A3M (open circles and dotted regression lines); the y-axis shows the result of individual bisulfite sequencing. The legends show the Pearson correlation coefficients. (PDF) [file pone.0041528.s003.pdf]

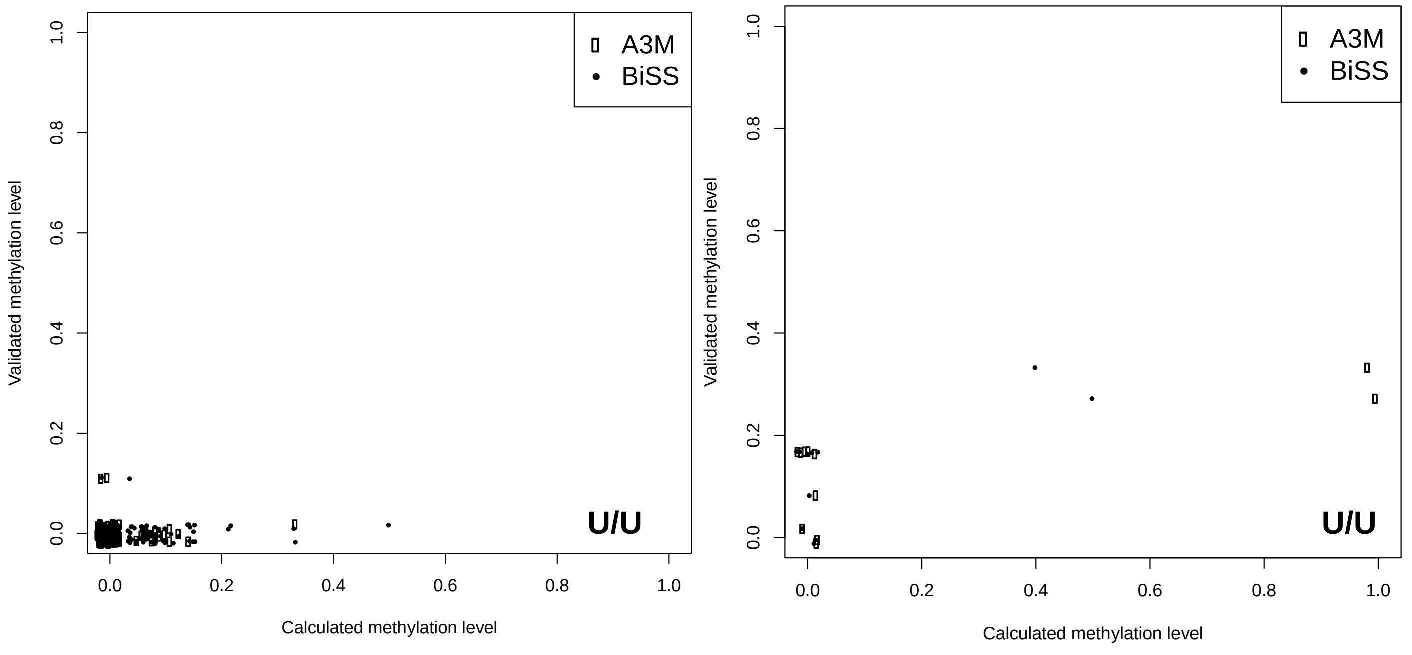

Dinh et al. Supplementary Figure 3

Validation by individual bisulfite sequencing. The plot shows the correlation between calculated and validated methylation levels (C/(C+T)) from a region selected for disagreement between BiSS (calling it unmethylated) and A3M (calling it methylated). Each point represents one cytosine position. The x-axis corresponds to the methylation levels calculated from either BiSS (filled circles and black regression lines) or A3M (open circles and dotted regression lines); the y-axis shows the result of individual bisulfite sequencing. The legends show the Pearson correlation coefficients.
